# Metabolic and Volumetric Alterations in the Basal Ganglia and the Cerebellum in Dopa‐Responsive Dystonia in Symptomatic and Asymptomatic *GCH1* Mutation Carriers

**DOI:** 10.1002/mds.70332

**Published:** 2026-04-26

**Authors:** Jannik Prasuhn, Leon van Well, Marta M. Pokotylo, Feline Hamami, Joke‐Lina Aßmann, Katja Lohmann, Maximilian G. Ködderitzsch‐Mertins, Julia Henkel, Jan Uter, Alexander Münchau, Christine Klein, Anne Weissbach, Norbert Brüggemann

**Affiliations:** ^1^ Section of Movement Disorders, Department of Neurology, University Medical Center Schleswig‐Holstein (UKSH) Campus Lübeck Lübeck Germany; ^2^ Institute of Neurogenetics University of Lübeck Lübeck Germany; ^3^ Center for Brain, Behavior and Metabolism University of Lübeck Lübeck Germany; ^4^ Department of Neurology Johns Hopkins University School of Medicine Baltimore Maryland USA; ^5^ F.M. Kirby Research Center for Functional Brain Imaging Kennedy Krieger Institute Baltimore Maryland USA; ^6^ Institute of Systems Motor Science, Center for Rare Diseases University of Lübeck Lübeck Germany; ^7^ Center of Rare Diseases, University‐Medical Center Schleswig‐Holstein (UKSH) Campus Lübeck Lübeck Germany

**Keywords:** Dystonia, GCH1, dopa‐responsive dystonia, metabolic imaging, mitochondria

## Abstract

**Background:**

Dopa‐responsive dystonia is caused by pathogenic variants in the *GCH1* gene. Although its clinical features and reduced penetrance are known, in vivo metabolic and structural alterations in symptomatic (sMC) and asymptomatic mutation carriers (aMC) remain poorly understood.

**Objectives:**

The aims were to characterize the volumetric and neurometabolic brain changes in *GCH1* mutation carriers (MC) and explore their relationship with clinical severity.

**Methods:**

We studied 20 sMCs, 5 aMCs, and 25 mutation‐free healthy controls (HC) using volumetric magnetic resonance imaging (MRI) combined with ^31^phosphorus magnetic resonance spectroscopy imaging (^31^P‐MRSI) of the basal ganglia and cerebellum. Analysis of covariance (ANCOVA) was used for group comparisons, and correlations were assessed using clinical symptom severity rating scales.

**Results:**

Volumetric analyses revealed enlarged globus pallidus (16.6%, *P* = 0.0010) and putamen (7.2%, *P* = 0.0310) volumes in sMCs and increased cerebellar gray matter in aMCs (8.0%, *P* = 0.0500). Nicotinamide adenine dinucleotide (NAD) levels were significantly reduced in the basal ganglia of carriers (NAD/Pi [inorganic phosphate]: −14.7%, *P* = 0.0460; NAD/ATP‐α: −15.5%, *P* = 0.0180). In the cerebellum, aMCs demonstrated elevated high‐energy phosphate ratios ([ATP‐α + PCr]/Pi: 23.7%, *P* = 0.0170; ATP‐α/Pi: 21.3%, *P* = 0.0460; PCr [phosphocreatine]/Pi: 25.2%, *P* = 0.0090) compared with sMCs and HCs. Smaller cerebellar volumes correlated with greater dystonia severity (Burke‐Fahn‐Marsden Dystonia Rating Scale, ρ = −0.557, *P* = 0.0133), whereas lower basal ganglia NAD ratios correlated with higher Movement Disorder Society‐Unified Parkinson's Disease Rating Scale, Part III (ρ = −0.527, *P* = 0.0204), and Toronto Western Spasmodic Torticollis Rating Scale scores (ρ = −0.475, *P* = 0.0398).

**Conclusions:**

Volumetric MRI and ^31^P‐MRSI reveal region‐ and subgroup‐specific metabolic and structural alterations in *GCH1* MCs, linking basal ganglia vulnerability and cerebellar adaptation to clinical severity. © 2026 The Author(s). *Movement Disorders* published by Wiley Periodicals LLC on behalf of International Parkinson and Movement Disorder Society.

Dopa‐responsive dystonia (DRD) represents a clinically and genetically heterogeneous group of dystonia parkinsonism most frequently caused by autosomal‐dominant inherited mutations in the *GCH1* gene (DYT/PARK‐*GCH1*).^1^ The disorder, also known as Segawa syndrome, is characterized by early‐onset dystonia with significant diurnal fluctuations, additional parkinsonian features in some patients, and a robust and lasting therapeutic response to low doses of levodopa (l‐dopa).^2^

Mutations in *GCH1* impair the biosynthesis of tetrahydrobiopterin (BH4), an essential cofactor for tyrosine hydroxylase, the rate‐limiting enzyme in dopamine synthesis. The resulting reduction in dopamine availability particularly affects the striatum and related basal ganglia circuits, crucial for controlling movement.^3^, ^4^ Postmortem studies and biochemical analyses have demonstrated reduced dopamine content in striatal tissue from affected individuals, yet in vivo markers of these metabolic disturbances remain scarce.^3^ Moreover, *GCH1* mutations exhibit incomplete penetrance, with some carriers developing dystonia or parkinsonism (symptomatic mutation carriers [sMC]), whereas others remain clinically unaffected (asymptomatic mutation carriers [aMC]).^1^ This variability is also present at the intrafamilial level, highlighting the importance of compensatory mechanisms that may mitigate the functional consequences of impaired dopamine synthesis.

There is increasing evidence that dystonia is associated not only with basal ganglia dysfunction but also with alterations in the cerebellum and cerebello‐thalamo‐striatal networks.^4^, ^5^, ^6^ Neuroimaging and animal studies in isolated and combined dystonias have suggested that the cerebellum may exert a modulatory or partially compensatory role.^5^, ^7^ Whether similar mechanisms are present in DRD, particularly in aMCs, remains an open question.

Advanced neuroimaging offers a unique opportunity to investigate these issues in vivo. ^31^Phosphorus magnetic resonance spectroscopy imaging (^31^P‐MRSI) allows the quantification of high‐energy phosphate (HEP) metabolites such as alpha‐adenosine triphosphate (ATP‐α) and phosphocreatine (PCr), as well as nicotinamide adenine dinucleotide (NAD), which are sensitive markers of cellular energy metabolism and mitochondrial function.^8^ Previous work in other movement disorders has demonstrated that alterations in NAD and HEP ratios reflect impaired bioenergetic capacity and may serve as early indicators of neurodegenerative or neurodevelopmental processes.^9^, ^10^, ^11^, ^12^ Structural magnetic resonance imaging (MRI) with automated volumetric segmentation provides robust measures of subcortical and cerebellar anatomy, which are primarily involved in dopaminergic motor circuits, allowing the detection of subtle structural alterations that may reflect neurodevelopmental or neurodegenerative changes, or compensatory plasticity.^13^

Despite the well‐characterized genetic and clinical profiles of DRD, systematic multimodal imaging studies investigating structural and neurometabolic correlates in *GCH1* mutation carriers (MC) are lacking. In this study, we set out to investigate the structural and neurometabolic features of *GCH1*‐associated DRD using a multimodal imaging approach that combined volumetric MRI and ^31^P‐MRSI of the basal ganglia and cerebellum in a cohort of sMCs, aMCs, and age‐ and sex‐matched, mutation‐free healthy controls (HC). Our aims were to determine whether alterations in NAD and HEP metabolism can be detected in vivo, to assess whether volumetric differences in basal ganglia and cerebellar structures accompany these metabolic features, and to explore the relationship between imaging markers and clinical features.

## Patients and Methods

### Recruitment and Clinical Characterization

The study protocol was reviewed and approved by the Ethics Committee of the University of Lübeck, and all procedures complied with the Declaration of Helsinki. Written informed consent was obtained from all participants prior to enrollment. Participants were identified through specialized inpatient and outpatient clinics and from existing research cohorts. Individuals were classified as either MCs or HCs. MCs were further subdivided into the sMC and aMC subgroups based on the presence or absence of manifest motor features. All participants underwent a standardized baseline assessment that included medical history, demographic information, and screening for contraindications to MRI. Genetic status was confirmed by genetic testing (sequencing and screening for copy number variants). Current medication use and previous neurological diagnoses were documented, with a detailed list of medications taken by sMCs presented in Table 1. Neurological examinations were performed by experienced specialists, video‐recorded, and rated in a blinded manner.^14^, ^15^ Motor impairment was rated using the motor part of the Movement Disorder Society‐Unified Parkinson's Disease Rating Scale, Part III (MDS‐UPDRS‐III),^16^ and dystonia severity was assessed using the Burke–Fahn–Marsden Dystonia Rating Scale (BFMDRS motor/disability)^17^ and the Toronto Western Spasmodic Torticollis Rating Scale (TWSTRS).^18^

**TABLE 1:** Demographic and clinical characteristics of symptomatic and asymptomatic GCH1 mutation carriers compared with mutation‐free healthy controls

	Symptomatic *GCH1* mutation carriers	Asymptomatic *GCH1* mutation carriers	Mutation‐free healthy controls
n (male/female)	20 (2/18)	5 (3/2)	25 (5/20)
AAE (yr)	43.5 ± 17.3	53.0 ± 25.7	46.6 ± 19.6
AAO (yr)	10.7 ± 12.3	n/a	n/a
LEDD (mg/day)	282.8 ± 189.4	n/a	n/a
Medication			
l‐Dopa	19/20 (95%)	n/a	n/a
DA	4/20 (20%)	n/a	n/a
COMT‐I	6/20 (30%)	n/a	n/a
MAO‐B‐I	5/20 (25%)	n/a	n/a
BFMDRS‐I	3.9 ± 3.4	n/a	n/a
BFMDRS‐II	4.3 ± 5.2	n/a	n/a
TWSTRS	17.1 ± 10.9	n/a	n/a
MDS‐UPDRS‐III	8.47 ± 8.06	n/a	n/a

*Note*: Values are shown as mean ± standard deviation.

Abbreviations: AAE, age at examination; AAO, age at onset; n/a, not applicable; LEDD, levodopa equivalent daily dose; l‐dopa, levodopa; DA, dopamine agonist; COMT‐I, catechyl‐*O*‐methyltransferase inhibitor; MAO‐B‐I, monoamine oxidase type B inhibitor; BFMDRS‐I, Burke–Fahn–Marsden Dystonia Rating Scale (motor score); BFMDRS‐II, Burke–Fahn–Marsden Dystonia Rating Scale (disability score); TWSTRS, Toronto Western Spasmodic Torticollis Rating Scale; MDS‐UPDRS‐III, Movement Disorder Society‐Unified Parkinson's Disease Rating Scale, Part III.

### Neuroimaging Acquisition and Analyses

All imaging was conducted on a 3‐T Siemens MAGNETOM Skyra scanner (Siemens Heathineers, Erlangen, Germany) at the Center for Brain, Behavior, and Metabolism Core Facility. sMCs were investigated in the l‐dopa *on* state. All datasets were reviewed by neuroradiologists to exclude previously undiagnosed structural abnormalities. Only complete scans were considered for subsequent analyses.

#### T1‐Weighted Imaging

Structural data were acquired using a standardized three‐dimensional (3D) magnetization‐prepared rapid gradient‐echo (MPRAGE) sequence, with a total scan time of 5 minutes and 52 seconds. This protocol enabled isotropic imaging with coverage in sagittal, coronal, and axial planes, yielding sharp T1‐weighted contrast through magnetization preparation and efficient acquisition with rapid gradient echoes. Parallel imaging was applied with an acceleration factor of two, reducing scan time and minimizing motion‐related artifacts. Sequence parameters were as follows: voxel size = 0.8 × 0.8 × 0.8 mm^3^, field of view = 320 × 320 × 320 mm^3^, TR (repetition time) = 2300 ms, TE (echo time) = 2.43 ms, TI (inversion time) = 1100 ms, and flip angle = 8°.

#### ^31^Phosphorus Magnetic Resonance Spectroscopy Imaging

^31^P‐MRSI was performed using a double‐tuned quadrature head coil (^1^H/^31^P, RAPID Biomedical, Erlangen, Germany) and a 3D chemical‐shift imaging free‐induction decay sequence. Acquisition parameters were as follows: voxel size = 30 × 30 × 30 mm^3^, field of view = 240 × 240 × 240 mm^3^, TR = 2000 ms, TE = 2.3 ms, flip angle = 50°, sixfold weighted averaging, bandwidth = 2000 Hz, vector size = 1024, and Hamming filter width = 100%. Broadband proton decoupling (WALTZ‐4) was applied, whereas the nuclear Overhauser effect was disabled. Total scan time was 8 minutes and 4 seconds. Four voxels were placed in the basal ganglia covering the bilateral putamen and globus pallidus regions. Placement was standardized using anatomical landmarks such as the anterior commissure and the internal capsule. Two voxels in the cerebellar hemispheres were positioned lateral to the vermis and aligned relative to the fourth ventricle to maximize coverage of gray matter (GM) and minimize contamination from adjacent structures (Fig. S1). Shimming was adjusted manually using a slightly larger shim volume than the predefined volume of interest. We initially examined metabolite ratios separately for individual voxels. After the absence of hemispheric asymmetry or systematic spatial gradients, we calculated side‐averaged values using the anterior two voxels for the basal ganglia and two voxels from the cerebellum to reduce measurement variability and increase the signal‐to‐noise ratio.

#### Neuroimaging Postprocessing and Quantification

Volumetric analyses were performed using the volBrain platform.^19^ T1‐weighted MPRAGE images were preprocessed using denoising, inhomogeneity correction, and spatial normalization to Montreal Neurology Institute space. Total intracranial volume (TIV) was estimated, and tissue segmentation into GM, white matter, and cerebrospinal fluid compartments was performed. Regional volumetry was derived from the volBrain submodules, providing automated parcellation of subcortical structures (caudate nucleus, putamen, globus pallidus) and total cerebellar volume and cerebellar GM. All volumetric measures were quality controlled through visual inspection of segmentation outputs. For group comparisons, TIV‐normalized data were used to account for head size variability. Non‐TIV‐normalized volumetric values were retained for correlation analyses with ^31^P‐MRSI‐derived metabolite levels. As ^31^P‐MRSI voxels were placed in each participant's native space, we performed correlations between volumetric MRI and MRSI‐derived metabolite measures, using individual outputs to ensure consistency between modalities. Spectra were processed using a Siemens workstation with the Syngo MR ER11 software package, as in our previous studies.^9^, ^20^, ^21^, ^22^ Preprocessing steps included Fourier transformation, zero filling, frequency and phase correction, curve fitting, and baseline adjustment. Ratios normalized to inorganic phosphate (Pi) were calculated for ATP‐α, PCr, and the combined (ATP‐α + PCr), with (ATP‐α + PCr)/Pi defined as the primary outcome. ATP‐α/Pi and PCr/Pi served as secondary variables. Additionally, we calculated NAD/Pi and NAD/ATP‐α as primary outcome measures to assess NAD metabolism. To ensure robustness, nonnormalized levels were additionally analyzed in an exploratory fashion.

### Statistical Analyses

All statistical procedures were conducted in *Jamovi* (version *2.3.21.0*). Descriptive statistics summarize demographic and clinical variables across subgroups, reported as means and standard deviations. Distributional assumptions were examined using quantile–quantile plots and formally tested using the Shapiro–Wilk test. Group comparisons of neuroimaging outcomes were performed using analysis of covariance (ANCOVA). Dependent variables included both normalized and nonnormalized metabolite levels and volumetric measures. The group assignment served as the fixed factor, whereas age, sex, and TIV were included as covariates to account for potential confounding effects. Analyses were first carried out in a two‐group design, contrasting all MCs with HCs, and subsequently extended to a three‐group design that distinguished between sMCs, aMCs, and HCs. When ANCOVA yielded significant results, post hoc pairwise comparisons were performed using Bonferroni correction to control for type I error. Finally, correlation analyses were performed to explore associations between ^31^P‐MRSI‐derived metabolite values, nonnormalized volumetric measures of basal ganglia and cerebellar structures, and clinical severity scores (BFMDRS, TWSTRS, MDS‐UPDRS‐III). Spearman's rank correlation coefficients were reported.

## Results

### Demographics and Clinical Characteristics

The study included 50 participants: 20 sMCs, 5 aMCs, and 25 HCs (Table 1; Table S1). Mean age was comparable across groups (sMC: 43.5 ± 17.3, aMC: 53.0 ± 25.7, and HC: 46.6 ± 19.6 years). In sMCs, the average age at onset was 10.7 ± 12.3 years, and the average levodopa equivalent daily dose (LEDD) was 282.8 ± 189.4 mg/day. Clinical scores in sMCs in the *on* state suggested a sufficient degree of symptom control with a BFMDRS mobility score of 3.9 ± 3.4, and a BFMDRS severity score of 4.3 ± 5.2, TWSTRS scores of 18.8 ± 10.6, and mild parkinsonian symptoms with an MDS‐UPDRS‐III score of 8.5 ± 8.0.

### Increased Basal Ganglia Volumes in GCH1 Mutation Carriers

**FIG. 1:**
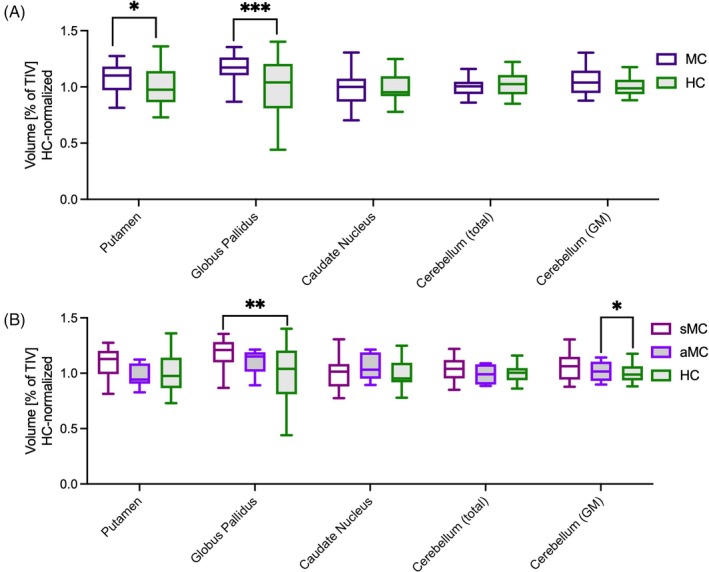
Group comparisons of subcortical and cerebellar volumetric measures across symptomatic and asymptomatic *GCH1* mutation carriers (MC) compared with HCs. Brain volumes are presented as a percentage of total intracranial volume and normalized to the healthy control mean. (**A**) A comparison of MCs (in purple) and HCs (in green). (**B**) A comparison of sMCs (in pink), aMCs (in purple), and HCs (in green). The significant group differences are indicated by brackets (***P< 0.001, **P < 0.01, *P < 0.05). aMC, asymptomatic mutation carriers; GM, gray matter; HC, mutation‐free healthy controls; sMC, symptomatic mutation carriers; TIV, total intracranial volume. [Color figure can be viewed at wileyonlinelibrary.com]

In the two‐group analysis of variance (ANOVA, MC vs. HC), basal ganglia volumes were higher in MCs than in HCs (Fig. 1; Table S2). Putamen volume was 7.2% higher (*F*_(1,46)_ = 4.932, *P* = 0.0310, η^2^ = 0.060), whereas globus pallidus volume was 16.6% higher in MCs than HCs (*F*_(1,46)_ = 11.950, *P* = 0.0010, η^2^ = 0.143). Caudate nucleus volumes did not differ (*F*_(1,46)_ = 0.556, *P* = 0.4600, η^2^ = 0.008). Both cerebellar total and cerebellar GM volumes were similar between MCs and HCs (cerebellar volume: *F*_(1,46)_ = 1.111, *P* = 0.2970, η^2^ = 0.021; cerebellar GM volume: *F*_(1,46)_ = 3.402, *P* = 0.0720, η^2^ = 0.061).

In the three‐group ANCOVA (sMC, aMC, HC), an increase of 18.3% in globus pallidus volume was most pronounced in sMCs (*F*_(2,45)_ = 6.090, *P* = 0.0050, η^2^ = 0.146; post hoc sMC vs. HC *P* = 0.0040; Table S3). Putamen and caudate nucleus volumes were similar across groups and did not differ significantly after correction (putamen: *F*_(2,45)_ = 2571, *P* = 0.0880, η^2^ = 0.064; caudate nucleus: *F*_(2,45)_ = 0.963, *P* = 0.3900, η^2^ = 0.027). Similarly, total cerebellar volume did not differ significantly across groups (*F*_(2,45)_ = 1.580, *P* = 0.2160, η^2^ = 0.059). Cerebellar GM significantly differed between groups (*F*_(2,45)_ = 3.290, *P* = 0.0460, η^2^ = 0.113), with 8.0% higher volume in aMCs compared to HCs (post hoc aMC vs. HC *P* = 0.0500).

### Reduced Nicotinamide Adenine Dinucleotide Ratios in the Basal Ganglia of GCH1 Mutation Carriers

**FIG. 2:**
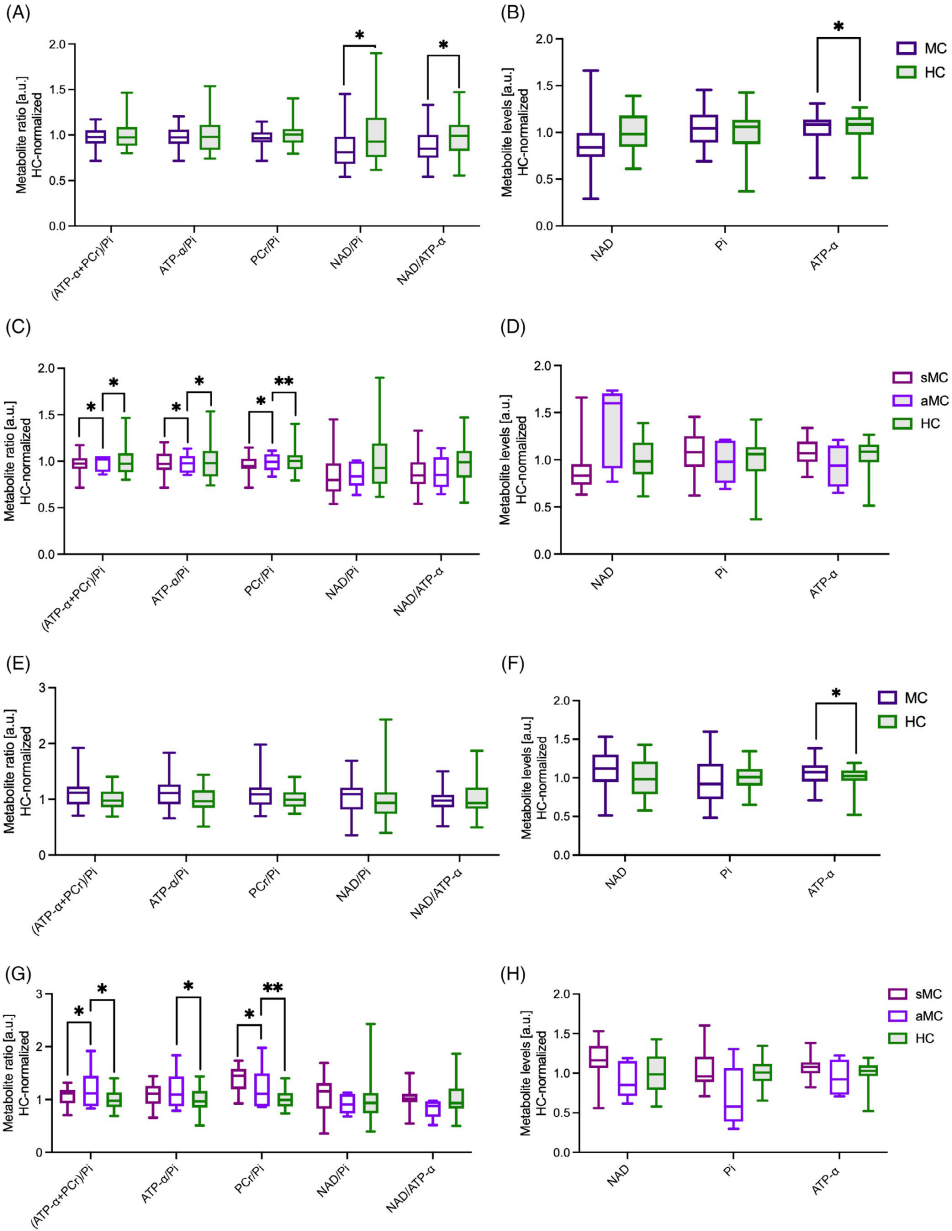
Group comparisons of ^31^phoshorus magnetic resonance spectroscopy–derived metabolite ratios in the basal ganglia and cerebellum across symptomatic and asymptomatic *GCH1* mutation carriers compared with HCs. Demonstrated side‐averaged values are derived from the (**A–D**) anterior two voxels in the basal ganglia and (**E–H**) two voxels in the cerebellum, expressed in arbitrary units and normalized to the healthy control mean. Panels A, B, E, and F present two‐group ANCOVA (analysis of covariance) comparing combined mutation carriers (sMCs and aMCs) in purple and HCs in green. Panels C, D, G, and H present three‐group ANCOVA comparing symptomatic (in pink), asymptomatic (in purple) *GCH1* mutation carriers, and HCs (in green). Statistically significant group differences are indicated by brackets (**P < 0.01, *P < 0.05). aMC, asymptomatic mutation carriers; ATP‐α, adenosine triphosphate alpha; a.u., arbitrary units; HC, mutation‐free healthy controls; NAD, nicotinamide adenine dinucleotide; PCr, phosphocreatine; Pi, inorganic phosphate; sMC, symptomatic mutation carriers. [Color figure can be viewed at wileyonlinelibrary.com]

In the two‐group ANCOVA (MC, HC), normalized ^31^P‐MRSI ratios of HEPs, such as (ATP‐α + PCr)/Pi (*F*_(1,45)_ = 0.542, *P* = 0.4650, η^2^ = 0.011), ATP‐α/Pi (*F*_(1,45)_ = 0.018, *P* = 0.7830, η^2^ = 0.002), and PCr/Pi (*F*_(1,45)_ = 1.521, *P* = 0.2240, η^2^ = 0.030), did not differ significantly between MCs and HCs (Fig. 2A,B; Table S4). NAD ratios were significantly reduced in carriers, specifically −14.7% in NAD/Pi (*F*_(1,45)_ = 4.199, *P* = 0.0460, η^2^ = 0.084) and −15.5% in NAD/ATP‐α (*F*_(1,45)_ = 5.989, *P* = 0.0180, η^2^ = 0.104) in MCs compared to HCs. Nonnormalized levels of NAD (−8.0%, *P* = 0.2360), Pi (6.2%, *P* = 0.1470), and ATP‐α (4.5%, *P* = 0.0950) differed between groups, although not statistically significant.

In the three‐group ANCOVA (sMC, aMC, HC), normalized HEP ratios of (ATP‐α + PCr)/Pi (*F*_(2,44)_ = 0.267, *P* = 0.7670, η^2^ = 0.011), ATP‐α/Pi (*F*_(2,44)_ = 0.042, *P* = 0.9590, η^2^ = 0.002), and PCr/Pi (*F*_(2,44)_ = 0.770, *P* = 0.4690, η^2^ = 0.031) did not differ significantly (Fig. 2C,D; Table 2). NAD ratios were lower in MCs; however, this difference was not statistically significant. NAD/Pi was 15.9% lower in sMCs and 10.2% lower in aMCs compared to HCs (*F*_(2,44)_ = 2.072, *P* = 0.1380, η^2^ = 0.085). NAD/ATP‐α was 15.2% lower in sMCs and 5.4% lower in aMCs compared to HCs (*F*_(2,44)_ = 2.953, *P* = 0.0630, η^2^ = 0.106). Similarly, nonnormalized levels of NAD (*F*_(2,44)_ = 0.952, *P* = 0.3940, η^2^ = 0.037), ATP‐α (*F*_(2,44)_ = 1.540, *P* = 0.2260, η^2^ = 0.039), and Pi (*F*_(2,44)_ = 1.112, *P* = 0.3380, η^2^ = 0.338) did not differ significantly, demonstrating numerically similar differences.

**TABLE 2:** Summarized results of three‐group ANCOVA of HEP metabolites in the basal ganglia and cerebellum of asymptomatic and symptomatic GCH1 mutation carriers and HCs

	sMC (n = 20)	aMC (n = 5)	HC (n = 25)	*P*_ANCOVA_	*P*_sMC‐aMC_	*P*_sMC‐HC_	*P*_aMC‐HC_
**Basal ganglia**							
(ATP‐α + PCr)/Pi	6.62 ± 0.71	6.60 ± 0.62	6.82 ± 0.98	0.7670	1.0000	1.0000	1.0000
ATP‐α/Pi	3.13 ± 0.36	3.03 ± 0.35	3.16 ± 0.58	0.9590	1.0000	1.0000	1.0000
PCr/Pi	3.49 ± 0.38	3.56 ± 0.38	3.66 ± 0.47	0.4690	1.0000	0.8930	1.0000
NAD/Pi	0.30 ± 0.07	0.32 ± 0.06	0.35 ± 0.10	0.1380	1.0000	0.1700	0.9980
NAD/ATP‐α	0.09 ± 0.02	0.11 ± 0.02	0.11 ± 0.02	0.0630	1.0000	0.0730	0.7350
NAD	58.7 ± 15.5	57.6 ± 20.1	63.6 ± 16.6	0.3940	1.0000	0.5390	1.0000
ATP‐α	619.6 ± 75.8	536.8 ± 135.1	577.2 ± 113.1	0.2260	1.0000	0.5000	0.5660
Pi	200.5 ± 35.3	177.6 ± 42.2	184.4 ± 35.9	0.3380	1.0000	0.6530	0.8750
**Cerebellum**							
(ATP‐α + PCr)/Pi	7.82 ± 1.43	9.88 ± 2.83	7.54 ± 1.47	**0.0017**	**0.0270**	1.0000	**0.0160**
ATP‐α/Pi	3.37 ± 0.76	4.09 ± 1.13	3.22 ± 0.72	**0.0460**	0.0710	1.0000	**0.0440**
PCr/Pi	4.45 ± 0.69	5.79 ± 1.74	4.32 ± 0.79	**0.0090**	0.0140	1.0000	**0.0080**
NAD/Pi	0.38 ± 0.12	0.38 ± 0.05	0.36 ± 0.15	0.6210	1.0000	1.0000	1.0000
NAD/ATP‐α	0.11 ± 0.02	0.09 ± 0.03	0.11 ± 0.03	0.6620	1.0000	1.0000	1.0000
NAD	66.8 ± 14.9	50.9 ± 16.8	60.9 ± 15.8	0.7510	1.0000	1.0000	1.0000
ATP‐α	599.0 ± 73.1	521.4 ± 124.6	551.6 ± 83.0	0.1330	1.0000	0.1430	1.0000
Pi	185.0 ± 41.1	136.6 ± 58.0	176.7 ± 32.6	0.0770	0.0740	0.9360	0.2270

*Notes*: Mean values (mean ± SD) of HEP metabolites (in arbitrary units) and metabolite ratios measured using ^31^phosphorus magnetic resonance spectroscopy imaging in the basal ganglia and the cerebellum. The table includes overall *P*‐values for the group effect (*P*_ANCOVA_) as well as post hoc comparisons (*P*_sMC‐aMC_, *P*_sMC‐HC_, and *P*_aMC‐HC_) between groups using Tukey's honestly significant difference post hoc testing. Significant results are presented in bold font.

Abbreviations: ANCOVA, analysis of covariance; HEP, high‐energy phosphate; HC, mutation‐free healthy control; sMC, symptomatic *GCH1* mutation carrier; aMC, asymptomatic *GCH1* mutation carrier; ATP‐α, alpha‐adenosine triphosphate; PCr, phosphocreatine; Pi, inorganic phosphate; NAD, nicotinamide adenine dinucleotide; SD, standard deviation.

### Increased High‐Energy Phosphate Ratios in the Cerebellum of Asymptomatic GCH1 Mutation Carriers

In the two‐group ANCOVA (MC, HC), normalized ^31^P‐MRSI ratios of HEPs were slightly higher in MCs but not statistically significant (Fig. 2E,F; Table S3). The combined (ATP‐α + PCr)/Pi was 9.1% higher in MCs (*F*_(1,45)_ = 1.276, *P* = 0.2650, η^2^ = 0.026), whereas ATP‐α/Pi and PCr/Pi were 9.3% (*F*_(1,45)_ = 1.036, *P* = 0.3140, η^2^ = 0.021) and 9.0% (*F*_(1,45)_ = 1.411, *P* = 0.2410, η^2^ = 0.029) higher in HCs. NAD ratios did not differ between groups: NAD/Pi was 4.1% higher (*F*_(1,45)_ = 0.019, *P* = 0.8900, η^2^ = 0.000), whereas NAD/ATP‐α was 2.7% lower in MCs (*F*_(1,45)_ = 0.761, *P* = 0.3880, η^2^ = 0.013). For nonnormalized levels, ATP‐α was 5.8% higher in MCs (*F*_(1,45)_ = 4.056, *P* = 0.0500, η^2^ = 0.055), whereas Pi was 0.8% lower in MCs (*F*_(1,45)_ = 0.0330, *P* = 0.8560, η^2^ = 0.001). NAD was 4.6% higher in MCs; this difference was not significant (*F*_(1,45)_ = 0.108, *P* = 0.7440, η^2^  = 0.000).

In the three‐group ANCOVA (sMC, aMC, HC), normalized HEP ratios showed a significant increase in aMCs relative to sMCs and HCs (Fig. 2G,H; Table 2). (ATP‐α + PCr)/Pi was 3.6% higher in aMCs than in HCs, and 30.9% higher in aMCs compared to sMCs (*F*_(2,44)_ = 4.456, *P* = 0.0170, η^2^ = 0.154; post hoc aMC vs. HC *P* = 0.0160, aMC vs. sMC *P* = 0.0270). ATP‐α/Pi was 27.1% higher in aMCs compared to HCs (*F*_(2,44)_ = 3.314, *P* = 0.0460, η^2^ = 0.117; post hoc aMC vs. HC *P* = 0.0440). PCr/Pi was 2.7% higher in aMCs compared to HCs, and 33.9% higher in aMCs compared to sMCs (*F*_(2,44)_ = 5.240, *P* = 0.0090, η^2^ = 0.179; post hoc aMC vs. HC *P* = 0.0080, aMC vs. sMC *P* = 0.0140). NAD ratios remained unaltered (NAD/Pi: *F*_(2,44)_ = 0.482, *P* = 0.6210, η^2^ = 0.015; NAD/ATP‐α: *F*_(2,44)_ = 0.417, *P* = 0.6620, η^2^ = 0.015). Nonnormalized levels pointed in the same direction, with no statistically significant differences (ATP‐α: *F*_(2,44)_ = 2.114, *P* = 0.1330, η^2^ = 0.060; Pi: *F*_(2,44)_ = 2.721, *P* = 0.0770, η^2^ = 0.091; NAD: *F*_(2,44)_ = 0.289, *P* = 0.7510, η^2^ = 0.009).

### Reduced Cerebellar Volumes and Lower NAD Ratios Are Associated with Greater Clinical Severity in Symptomatic GCH1 Mutation Carriers

**FIG. 3:**
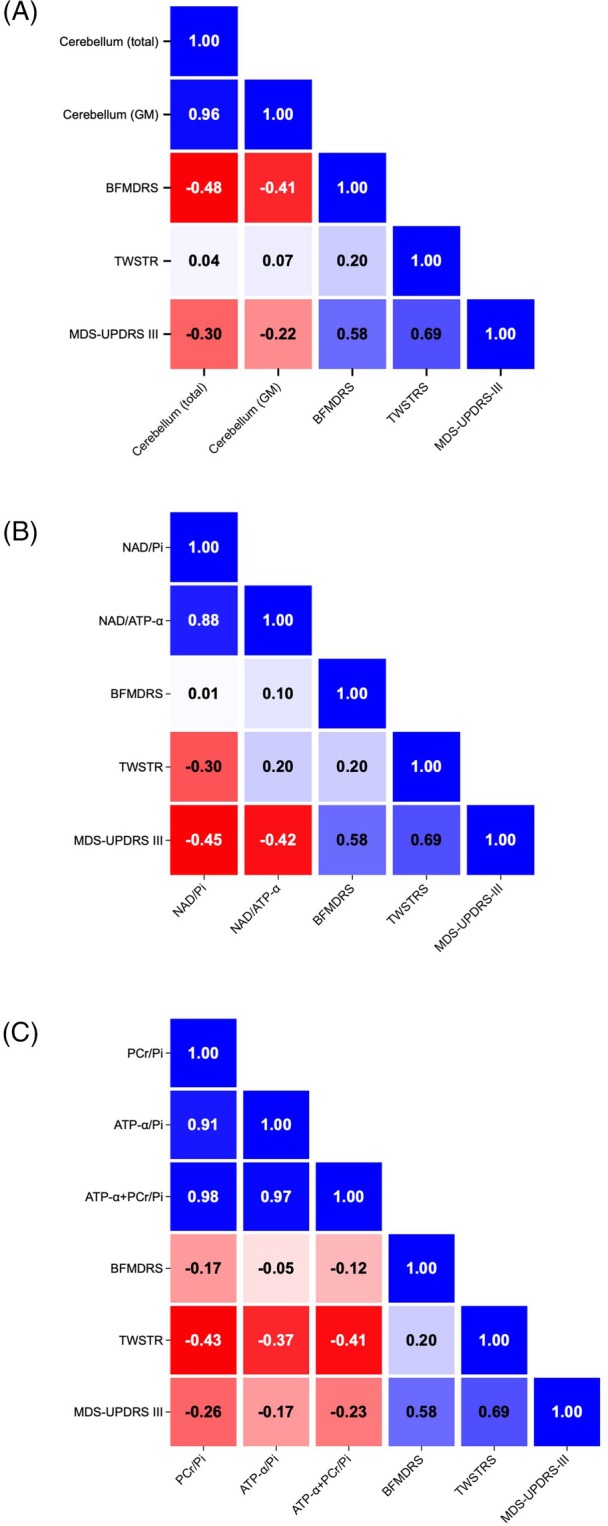
Correlation analyses of volumetric measures, ^31^phosphorus magnetic resonance spectroscopy imaging metabolite ratios, and clinical scores in symptomatic *GCH1* mutation carriers. The heat maps demonstrate the correlations between (**A**) brain structure volumes, (**B**) metabolite ratios in the basal ganglia, and (**C**) cerebellum, and clinical scores. ATP‐α, adenosine triphosphate alpha; BFMDRS, Burke–Fahn–Marsden Dystonia Rating Scale; GM, gray matter; MDS‐UPDRS‐III, Movement Disorder Society‐Unified Parkinson's Disease Rating Scale, Part III; NAD, nicotinamide adenine nucleotide; PCr, phosphocreatine; Pi, inorganic phosphate; TWSTRS, Toronto Western Spasmodic Torticollis Rating Scale. [Color figure can be viewed at wileyonlinelibrary.com]

Using nonnormalized volumetric and ^31^P‐MRSI measures, we observed no disease‐relevant associations in aMCs. However, smaller total cerebellar volume correlated with higher BFMDRS scores in sMCs (ρ = −0.557, *P* = 0.0133). Correlation analysis between ^31^P‐MRSI ratios and clinical scores revealed that lower basal ganglia NAD ratios were associated with more severe motor impairment (Fig. 3; Fig. S2). NAD/Pi correlated negatively with MDS‐UPDRS‐III scores (ρ = −0.527, *P* = 0.0204), whereas NAD/ATP‐α correlated negatively with TWSTRS scores (ρ = −0.475, *P* = 0.0398) and exhibited a trend with MDS‐UPDRS‐III (ρ = −0.422, *P* = 0.0720). Additionally, MDS‐UPDRS‐III scores correlated positively with both BFMDRS (disability score: ρ = 0.681, *P* = 0.0013) and TWSTRS (ρ = 0.494, *P* = 0.0315).

## Discussion

In this study, we combined volumetric MRI and ^31^P‐MRSI to characterize the structural and neurometabolic features of *GCH1*‐related DRD in vivo. Three main observations emerged: (1) basal ganglia volumes were larger in aMCs and sMCs, whereas cerebellar GM volumes were larger in aMCs compared to HCs only; (2) NAD ratios were consistently reduced in the basal ganglia of sMCs and aMCs; and (3) HEP ratios were increased in the cerebellum of aMCs. Importantly, reduced cerebellar volumes and lower basal ganglia NAD ratios were associated with greater clinical severity, linking imaging‐derived markers to the phenotype. Our data support the concept of cerebellar compensation in DRD. The increase in cerebellar HEP metabolism, particularly in aMCs, may reflect enhanced mitochondrial activity that counterbalances basal ganglia dysfunction. These findings are supported by prior work demonstrating that the cerebellum contributes to the pathophysiology of dystonia through altered cerebello‐thalamo‐striatal signaling.^4^, ^20^, ^23^, ^24^ In our cohort, increased cerebellar GM volume in aMCs further suggests structural plasticity, potentially underlying the absence of overt symptoms in this subgroup.

The reduced NAD ratios in the basal ganglia are consistent with a functional bioenergetic deficit. As NAD is a cofactor in BH4 regeneration, reduced NAD availability may reflect impaired or exhausted BH4 recycling, further exacerbating the dopaminergic deficit caused by the pathogenic *GCH1* variants.^3^, ^25^ Enlargement of the globus pallidus and putamen in MCs could reflect a compensatory response within striatal‐pallidal circuits.^26^, ^27^, ^28^ These findings need to be interpreted within the broader context of basal ganglia and cerebello‐thalamo‐cortical alterations described across several forms of monogenic dystonia, including aMCs with pathogenic *TOR1A* or *THAP1* variants. Structural and diffusion‐based imaging studies in these conditions have shown abnormalities within striatal‐pallidal circuits and in their connectivity with cerebellar and thalamo‐cortical pathways, typically reflecting network dysfunction and reorganization rather than pronounced striatal atrophy. Supporting the network model, enlargement of the globus pallidus and putamen in MCs may therefore represent a compensatory remodeling of striatal‐pallidal loops in response to chronic dopaminergic dysfunction, which would be consistent with the absence of striatal atrophy in patients with DRD and distinguishes it from neurodegenerative dystonia‐parkinsonism disorders, where progressive basal ganglia volume loss is prominent.^4^, ^29^, ^30^, ^31^, ^32^, ^33^ Together, these results suggest that *GCH1*‐related dystonia is characterized by a combination of basal ganglia vulnerability and cerebellar adaptation, with correlations between imaging markers and clinical severity further supporting its clinical relevance. Lower basal ganglia NAD ratios correlated with higher motor severity, whereas smaller cerebellar volumes were associated with more pronounced dystonic symptoms. Interestingly, higher cerebellar HEP ratios were observed in aMCs, suggesting a protective metabolic mechanism that may delay or prevent symptom onset.

Several limitations need to be considered. The cross‐sectional design precludes conclusions about the temporal evolution of NAD and HEP metabolism in MCs, necessitating longitudinal studies to determine whether reduced basal ganglia NAD ratios or elevated cerebellar HEP ratios precede, parallel, or follow clinical symptom manifestation, and whether they predict future progression or resilience. Furthermore, the observed volumetric changes would benefit from a longitudinal approach to confirm whether they are due to compensatory or other pathogenic events occurring over the course of the disease. Although we standardized ^31^P‐MRSI voxel placement using anatomical landmarks to maximize coverage of the target regions, the spatial resolution remained limited by the relatively large voxel size, which may introduce partial volume effects, particularly in small subcortical nuclei, and obscure finer‐grained regional differences. Furthermore, spectral resolution did not allow differentiation between oxidation states of NAD.^34^, ^35^ Although our cohort was relatively large for a rare disease, subgroup analyses, especially in aMCs, remain constrained by small sample sizes, limiting the statistical power to detect subtle effects. Therefore, the aMC‐derived findings should be interpreted with caution, and future studies should increase sample size to confirm this study's observations. Although our sMC participants were examined in the *on* medication state, the mean LEDD was low (282 mg) and lower than typically expected in Parkinson's disease. Therefore, we cannot completely exclude a potential influence of dopaminergic medication on the observed changes in HEP metabolism, but we consider it to be less pronounced than in Parkinson's disease studies and, in particular, compared with our previous pilot study, in which we performed an acute l‐dopa challenge (l‐dopa/benserazide 200/50 mg).^21^ Furthermore, only sMCs received medications; therefore, the findings from sMCs and aMCs are not directly comparable, and future studies employing *off*‐state measurements could help disentangle disease‐intrinsic from therapy‐related changes. However, such an experimental approach may be challenging in patients with mobile dystonia; yet, in some patients, if the analysis is performed in the morning, it is generally feasible. Finally, ^31^P‐MRSI cannot disentangle cellular sources of metabolic signals, the relative contributions of neuronal versus glial compartments, or mitochondrial versus glycolytic ATP production. Similarly, volumetric MRI captures macrostructural changes but does not directly inform on microstructural adaptations that may underlie the observed enlargements. Furthermore, our methodological approach was limited to assessing the basal ganglia and cerebellum, with limited insights into cortical and thalamic regions. Further studies should take this into consideration and assess the relationship between structural and neurometabolic correlates across whole‐brain and large‐scale association networks.

In summary, our findings provide novel in vivo evidence that *GCH1*‐related DRD is characterized by structural adaptations in both basal ganglia and cerebellum, reduced basal ganglia NAD ratios, and increased cerebellar HEP metabolism. These results support a model in which cerebellar mechanisms compensate for basal ganglia dysfunction and highlight imaging‐derived bioenergetic markers as promising tools for understanding variability in clinical expression. Longitudinal multimodal studies are warranted to determine the temporal dynamics of these changes and their potential role as biomarkers for disease stratification and therapeutic monitoring.

## Author Roles

J.P.: study conception and design, project supervision, data interpretation, manuscript drafting. L.W.: data acquisition, data analysis, manuscript revision. M.M.P.: data acquisition, formal analysis, visualization, manuscript revision. F.H.: data acquisition, data analysis, manuscript revision. J.‐L.A.: data acquisition, data curation, manuscript revision. K.L.: (genetic) data acquisition and interpretation, manuscript revision. M.G.K.‐M.: methodology development, data analysis, manuscript revision. J.H.: data acquisition, data interpretation, manuscript revision. J.U.: data curation, statistical analysis, manuscript revision. A.M.: clinical interpretation, manuscript revision. C.K.: conceptual discussion, clinical interpretation, manuscript revision. A.W.: patient recruitment, clinical phenotyping, manuscript revision. N.B.: study conception, clinical supervision, data interpretation, manuscript revision.

## Author Contributions

(1) Research Project: A. Conception, B. Organization, C. Execution; (2) Statistical Analysis: A. Design, B. Execution, C. Review and Critique; (3) Manuscript Preparation: A. Writing of the First Draft, B. Review and Critique.

J.P.: 1A, 1B, 2C, 3A.

L.v.W.: 1C, 2B, 3B.

M.M.P.: 1C, 2A, 2B, 3B.

F.H.: 1C, 2B, 3B.

J.L.A.: 1C, 2B, 3B.

K.L.: 1C, 2C, 3B.

M.G.K.‐M.: 1B, 2A, 2B, 3B.

J.H.: 1C, 2C, 3B.

J.U.: 1B, 2A, 2B, 3B.

A.M.: 1C, 2C, 3B.

C.K.: 1A, 2C, 3B.

A.W.: 1C, 2C, 3B.

N.B.: 1A, 1B, 2C, 3B.

## Full financial disclosures of all authors for the preceding 12 months

The authors report no financial relationships or activities within the past 12 months that could be perceived as influencing the submitted work.

## Financial Disclosures and Conflicts of Interest

Author disclosures are available in the Supporting Information.

## Supporting information


**Figure S1.** Region‐specific voxel placement for ^31^phosphorus magnetic resonance.
**Figure S2.** Correlation between volumetric magnetic resonance imaging and ^31^phosphorus magnetic resonance.
**Table S1.** Clinical and genetic characteristics of *GCH1* mutation carriers.
**Table S2.** Results of two‐group ANCOVA (analysis of covariance) of volumetric magnetic resonance.
**Table S3.** Results of three‐group ANCOVA (analysis of covariance) of volumetric magnetic resonance.
**Table S4.** Summarized results of two‐group ANCOVA (analysis of covariance) of high energy.

## Data Availability

The data that support the findings of this study will be made available upon reasonable request from the corresponding author.
