# Supplementary material for: Metabolic and Volumetric Alterations in the Basal Ganglia and the Cerebellum in Dopa‐Responsive Dystonia in Symptomatic and Asymptomatic GCH1 Mutation Carriers
Source: Mov Disord. 2026 Apr 26;41(7):1716–26. doi: 10.1002/mds.70332 (PMC13387918; doi:10.1002/mds.70332)
Supplement: Supplementary file 1 — Figure S1. Region‐specific voxel placement for 31phosphorus magnetic resonance. Figure S2. Correlation between volumetric magnetic resonance imaging and 31phosphorus magnetic resonance. Table S1. Clinical and genetic characteristics of GCH1 mutation carriers. Table S2. Results of two‐group ANCOVA (analysis of covariance) of volumetric magnetic resonance. Table S3. Results of three‐group ANCOVA (analysis of covariance) of volumetric magnetic resonance. Table S4. Summarized results of two‐group ANCOVA (analysis of covariance) of high energy. [file MDS-41-1716-s001.pdf]

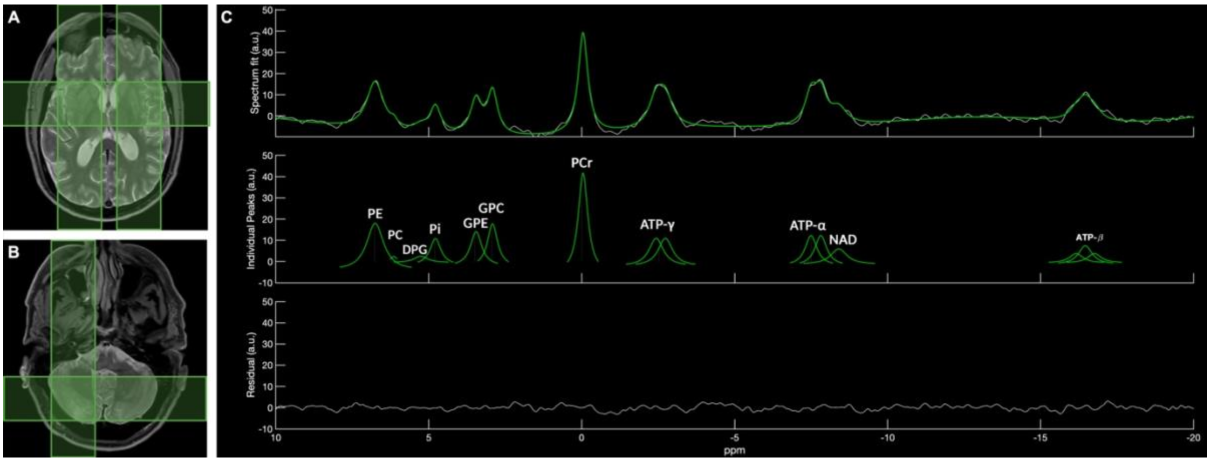

Supplementary Figure 1. Region-specific voxel placement for ^{31}phosphorus magnetic resonance spectroscopy imaging in the basal ganglia and cerebellum with a representative spectrum and corresponding postprocessing output. In panels A and B, we demonstrate anatomical localization of the acquisition voxel in the basal ganglia (A) and cerebellum (B) on axial sections of the T_2-weighted magnetic resonance images. The top section of panel C demonstrated the measured spectrum in white) overlaid with the fitted model (in green). The middle section displays the individual metabolite peaks, and the bottom section demonstrates the residuals following spectral fitting. ATP-α, -β, -γ = adenosine triphosphate-alpha, -beta, -gamma; a.u. = arbitrary units; DPG = diphosphoglycerate; GPC = glycerophosphocholine; GPE = glycerophosphoethanolamine; NAD = nicotinamide adenine dinucleotide; PC = phosphocholine; PCr = phosphocreatine; PE = phosphoethanolamine; Pi = inorganic phosphate; ppm = particles per million.

Supplementary Table 1. Clinical and genetic characteristics of *GCH1* mutation carriers.

sex	mutation	AAO [years]	AAD [years]	AAE [years]	LEDD [mg/day]	BFMDRS (Disability)	BFMDRS (Motor)	TWSTRS	MDS-UPDRS-III
m	c.631_632del; p.Met211Valfs*38	44	41	57	50	0.5	0	10.0	0
f	c.631_632del; p.Met211Valfs*38	5	11	27	50	0.0	1	6.0	0
f	c.571G>A; p.Val191Tyr	4	36	51	750	0.5	16	47.5	18
m	*c.126dupC; p.Glu43Argfs20**	*n/a*	*n/a*	*81*	*200*	*2.0*	*3*	*22.8*	*0*
m	c.488T>G; p.Leu163Arg	49	58	60	320	4.0	1	13.0	8
f	c.(1-?_736+?)del; p.0?	9	58	58	400	1.0	3	21.8	2
f	c.(1-?_736+?)del; p.0?	4	5	33	300	7.0	0	21.0	7
f	c.488T>G; p.Leu163Arg	6	10	32	435	2.5	0	9.3	3
f	c.262C>G; p.Arg88Gly	11	11	40	200	6.5	1	17.0	22
m	*c.626+2dupT; p.?*	*n/a*	*n/a*	*81*	*0*	*8.0*	*4*	*17.8*	*29*
f	c.626+2dupT; p.?	6	22	55	300	5.0	4	14.0	3
f	c.626+2dupT; p.?	8	30	57	300	3.5	11	28.8	12
f	c.680C>A; p.Thr227Asn	8	31	63	400	14.0	5	13.0	14
f	*c.680C>A; p.Thr227Asn*	*n/a*	*n/a*	*29*	*0*	*5.0*	*0*	*12.0*	*0*
f	c.181G>T; p.Glu61*	7	55	84	200	8.0	14	37.0	31
f	*c.607G>A; p.Gly203Arg*	*n/a*	*n/a*	*19*	*0*	*4.0*	*0*	*0.0*	*0*
m	c.607G>A; p.Gly203Arg	0	18	20	250	0.0	11	31.0	12
f	*c.607G>A; p.Gly203Arg*	*n/a*	*n/a*	*55*	*0*	*16.0*	*2*	*0.0*	*0*
f	c.607G>A; p.Gly203Arg	6	14	19	400	3.0	11	23.0	3
f	c.671A>G; p.Lys224Arg	13	52	55	150	5.0	1	4.0	11
f	c.(1-?_736+?)del; p.0?	3	21	47	700	n/a	n/a	n/a	n/a
f	c.527G>A; p.Ser176Asn	13	13	37	100	0.0	0	13.0	0
f	c.532A>G; p.Arg178Gly	7	24	28	100	6.0	2	17.0	4
f	c.532A>G; p.Arg178Gly	7	21	25	100	3.0	1	14.0	4
f	c.571G>A; p.Val191Tyr	5	5	21	150	4.0	0	17.0	7

The table comprises both asymptomatic (in italics) and symptomatic *GCH1* mutation carriers. AAO = age at onset, AAD = age at diagnosis, AAE = age at examination, LEDD = levodopa equivalent daily dose, HY = Hoehn & Yahr stage, BFMDRS I = Burke-Fahn-Marsden Dystonia Rating Scale (motor section), BFMDRS-II = Burke-Fahn-Marsden Dystonia Rating Scale (disability section), TWSTRS = Toronto Western Spasmodic Torticollis Rating Scale, MDS-UPDRS-III = Movement Disorder Society-Unified Parkinson's Disease Rating Scale, part III, n/a = not available.

Supplementary Table 2. Results of two-group ANCOVA analysis of volumetric magnetic resonance imaging in *GCH1* mutation carriers and mutation-free healthy controls.

	**MC** (n = 25)	**HC** (n = 25)	**p_{ANCOVA}**
Putamen	0.58 ± 0.07	0.54 ± 0.09	**0.0031**
Globus pallidus	0.18 ± 0.02	0.17 ± 0.04	**0.0001**
Caudate nucleus	0.49 ± 0.07	0.50 ± 0.06	0.4600
Cerebellar volume	9.77 ± 0.97	0.95 ± 0.71	0.2970
Cerebellar GM	7.99 ± 0.88	7.63 ± 0.55	0.072

Mean ± SD values of basal ganglia and cerebellar volumes expressed as %TIV. The table includes overall p-values for the group effect (p_{ANCOVA}). Significant results are printed in bold. GM = gray matter; HC = mutation-free healthy controls; MC = *GCH1* mutation carriers; TIV = total intracranial volume.

Supplementary Table 3. Results of three-group ANCOVA analysis of volumetric magnetic resonance imaging in symptomatic and asymptomatic *GCH1* mutation carriers and mutation-free healthy controls.

	**sMC** (n = 20)	**aMC** (n = 5)	**HC** (n = 25)	$\mathbf{p_{ANCOVA}}$	$\mathbf{p_{sMC\text{-}aMC}}$	$\mathbf{p_{sMC\text{-}HC}}$	$\mathbf{p_{aMC\text{-}HC}}$
Putamen	0.59 ± 0.07	0.54 ± 0.08	0.54 ± 0.09	0.0880	1.0000	0.0880	1.0000
Globus pallidus	0.20 ± 0.02	0.19 ± 0.02	0.17 ± 0.04	**0.0050**	1.0000	**0.0040**	0.5330
Caudate nucleus	0.50 ± 0.07	0.45 ± 0.06	0.50 ± 0.06	0.3900	0.7470	1.0000	0.5170
Cerebellar volume	9.72 ± 0.96	9.98 ± 1.08	9.53 ± 0.71	0.2160	0.4820	1.0000	0.2470
Cerebellar GM	7.92 ± 0.88	8.24 ± 0.93	7.63 ± 0.55	**0.0460**	0.2660	0.7710	**0.0500**

Mean ± SD values of basal ganglia and cerebellar volumes expressed as %TIV. The table includes overall p-values for the group effect (p_{ANCOVA}) as well as post hoc comparisons ($p_{sMC\text{-}aMC}$, $p_{sMC\text{-}HC}$, and $p_{aMC\text{-}HC}$) between groups using Tukey's Honestly Significant Difference post-hoc testing. Significant results are printed in bold. aMC = asymptomatic *GCH1* mutation carriers; GM = gray matter; HC = mutation-free healthy controls; sMC = symptomatic *GCH1* mutation carriers; TIV = total intracranial volume.

Supplementary Table 4. Summarized results of two-group ANCOVA analysis of high-energy phosphate metabolites in the basal ganglia and cerebellum of asymptomatic and symptomatic *GCH1* mutation carriers and mutation-free healthy controls.

	**MC** (n = 25)	**HC** (n = 25)	p_{ANCOVA}
**Basal ganglia**			
(ATP-α + PCr)/Pi	6.61 ± 0.68	6.82 ± 0.98	0.4650
ATP-α/Pi	3.11 ± 0.35	3.16 ± 0.58	0.7830
PCr/Pi	3.50 ± 0.37	3.66 ± 0.47	0.2240
NAD/Pi	0.30 ± 0.07	0.35 ± 0.10	**0.0460**
NAD/ATP-α	0.10 ± 0.02	0.11 ± 0.02	**0.0180**
NAD	58.5 ± 16.0	63.6 ± 16.6	0.2360
ATP-α	603.1 ± 93.5	577.2 ± 113.1	0.0950
Pi	195.9 ± 37.0	184.4 ± 35.9	0.1470
**Cerebellum**			
(ATP-α + PCr)/Pi	3.52 ± 0.09	3.22 ± 0.72	0.3140
ATP-α/Pi	4.71 ± 1.09	4.32 ± 0.79	0.2410
PCr/Pi	0.38 ± 0.11	0.36 ± 0.15	0.8900
NAD/Pi	0.11 ± 0.02	0.11 ± 0.03	0.3880
NAD/ATP-α	63.7 ± 16.3	60.9 ± 15.8	0.7440
NAD	583.5 ± 88.4	551.6 ± 83.0	**0.0500**
ATP-α	175.3 ± 47.9	176.7 ± 32.6	0.8560
Pi	3.52 ± 0.09	3.22 ± 0.72	0.3140

Mean ± SD values of high-energy phosphate metabolites (in arbitrary units) and metabolite ratios measured using ^{31}phosphorus magnetic resonance spectroscopy imaging in the basal ganglia and the cerebellum. The table includes overall p-values for the group effect (p_{ANCOVA}). Significant results are printed in bold. aMC = asymptomatic *GCH1* mutation carriers; ATP-α = alpha-adenosine triphosphate; HC = mutation-free healthy controls; NAD = nicotinamide adenine dinucleotide; PCr = phosphocreatine; Pi = inorganic phosphate; sMC = symptomatic *GCH1* mutation carriers.

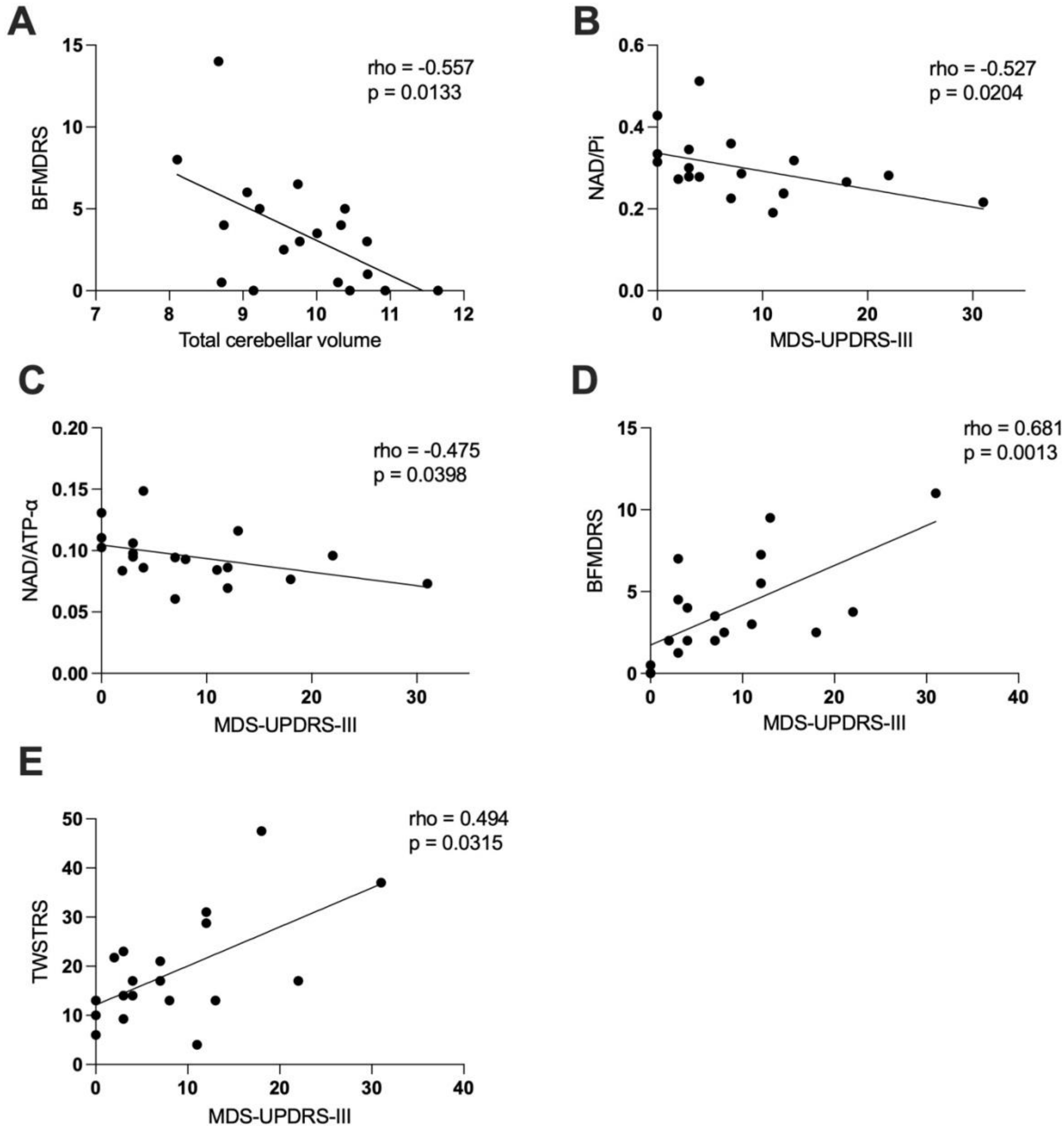

Supplementary Figure 2. Correlation between volumetric, [31]phosphorus magnetic resonance spectroscopy imaging measures and clinical scores in symptomatic *GCH1* mutation carriers. Scatter plots were presented to demonstrate the results of the correlation analysis, with Spearman's rank correlation coefficient (rho) and nominal p-values for each test. ATP-α = adenosine triphosphate-alpha; BFMDRS = Burke-Fahn-Marsden Dystonia Rating Scale; MDS- UPDRS-III = Movement Disorder Society Unified Parkinson's Disease Rating Scale III; NAD = nicotinamide adenine dinucleotide; Pi = inorganic phosphate; TWSTRS = Toronto Western Spasmodic Torticollis Rating Scale.
